# Supplementary material for: Candidate silencer elements for the human and mouse genomes
Source: Nat Commun. 2020 Feb 26;11:1061. doi: 10.1038/s41467-020-14853-5 (PMC7044160; doi:10.1038/s41467-020-14853-5)
Supplement: Supplementary file 2 — Supplementary Information [file 41467_2020_14853_MOESM2_ESM.pdf]

Supplemental Information

Candidate silencer elements for the human and mouse genomes

Naresh Doni Jayavelu[1], Ajay Jajodia[1], Arpit Mishra[1], R. David Hawkins[1,*]

Supplementary Figures

Supplementary Figure 1: Overview of uncharacterized CREs
Supplementary Figure 2: Functional testing of activity of uncharacterized CREs in K562 cells
Supplementary Figure 3: SVM model candidate silencer elements predictions
Supplementary Figure 4: Characteristics of candidate silencer elements
Supplementary Figure 5: 3D Genome interactions of candidate silencer elements
Supplementary Figure 6: Candidate silencer elements are enriched for GWAS SNPs and can act as enhancers in other cell types

Supplementary Tables

Supplementary Data 1: Overview of cell types and count of uncharacterized CREs per cell type
Supplementary Data 2: MPRA tested uncharacterized *cis*-regulatory elements along with control elements and their activity levels
Supplementary Data 3: Reporter assays testing silencer elements along with control elements and their activity levels
Supplementary Data 4: Primer and gblock sequences used in CRISPR-Cas9 experiments
Supplementary Data 5: SVM model identified candidate silencer elements

Supplementary Figure 1

a

Species	Consortium	Number of Cell types	Total uncharacterized CREs
Human	Roadmap ENCODE	82	2,315,105
Mouse	ENCODE	22	1,299,866

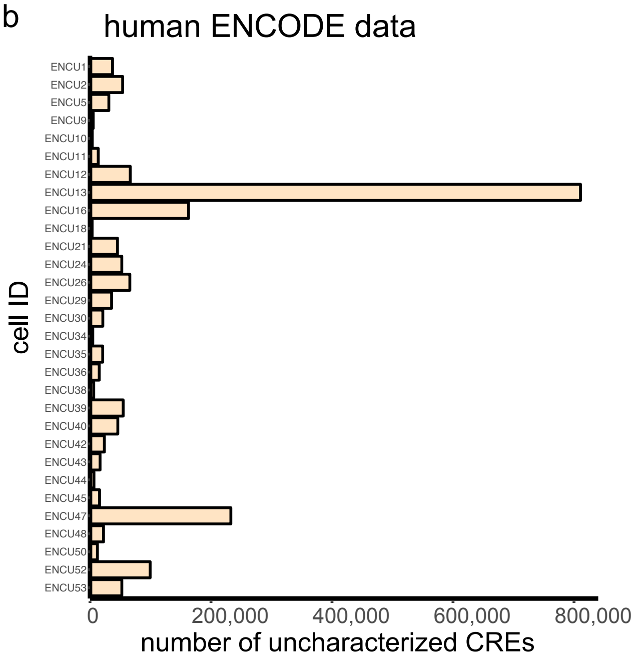

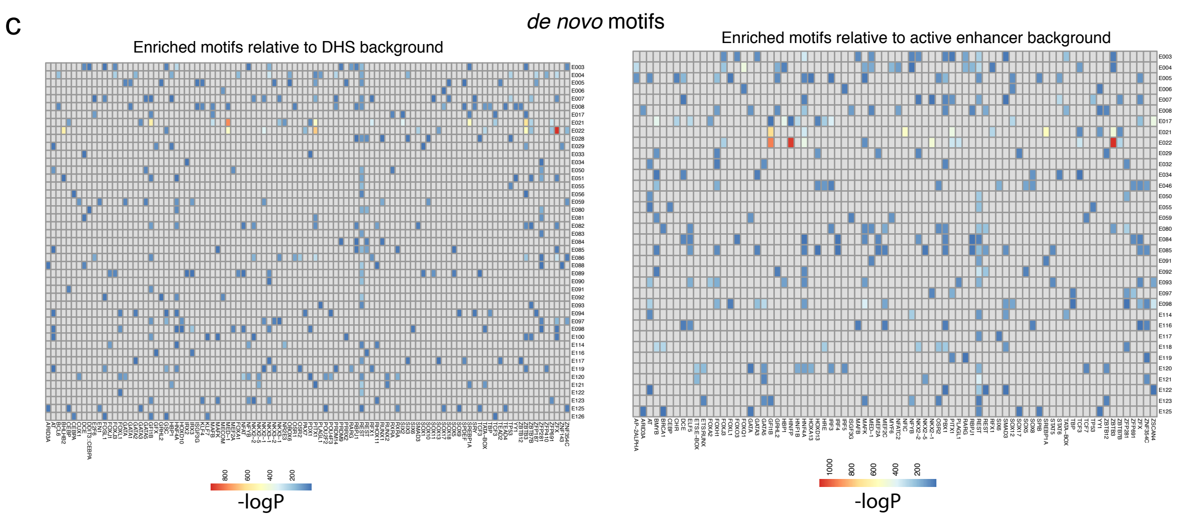

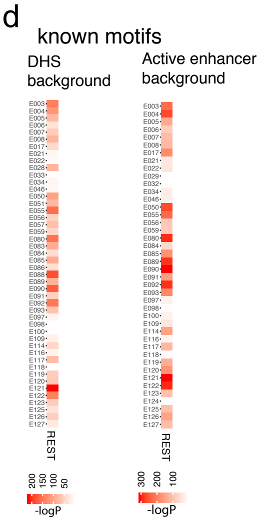

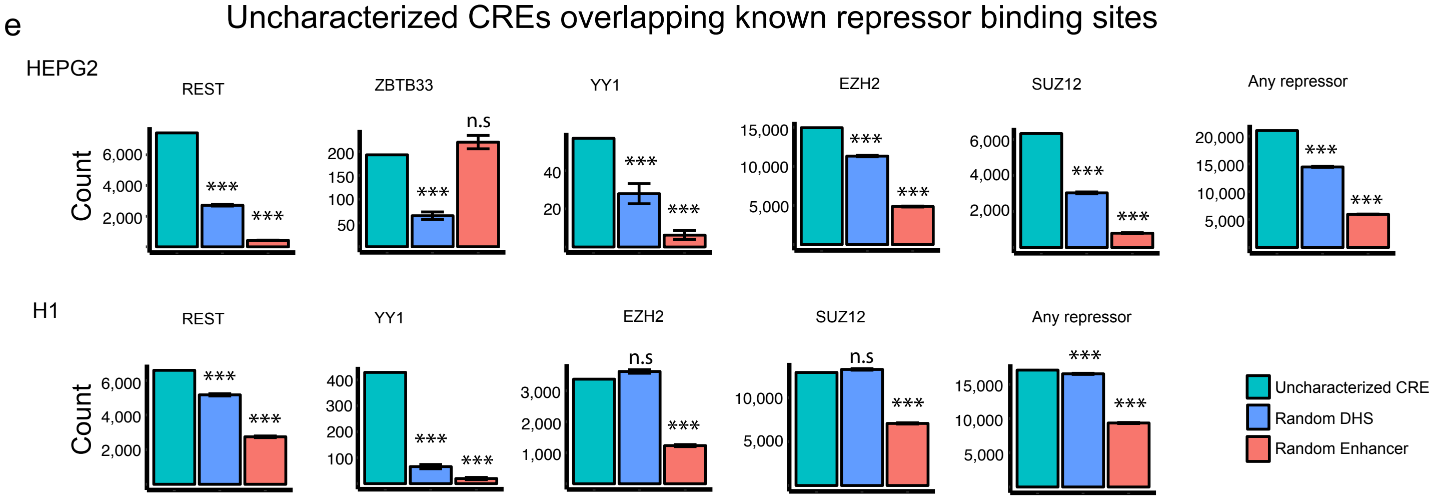

Supplementary Figure 1: Overview of uncharacterized CREs. (a) Count of total and cell-type specific uncharacterized CREs identified across the human and mouse genomes. (b) Bar plot presenting the count of uncharacterized CREs across different cell types and tissues in human from the ENCODE consortium. Heatmap of enrichment (-log(P-value)) of *de novo* TF motifs (c) and known TF motifs (d) at uncharacterized CREs in comparison to DHS and enhancer backgrounds. (e) Bar plots presenting the count of uncharacterized CREs, random DHS and random enhancers at known repressor TFBS based on ChIP-seq data in HEPG2 and H1 cell types. *** indicates permutation test P-value < 0.0001 and n.s. denotes not significant.

Supplementary Figure 2

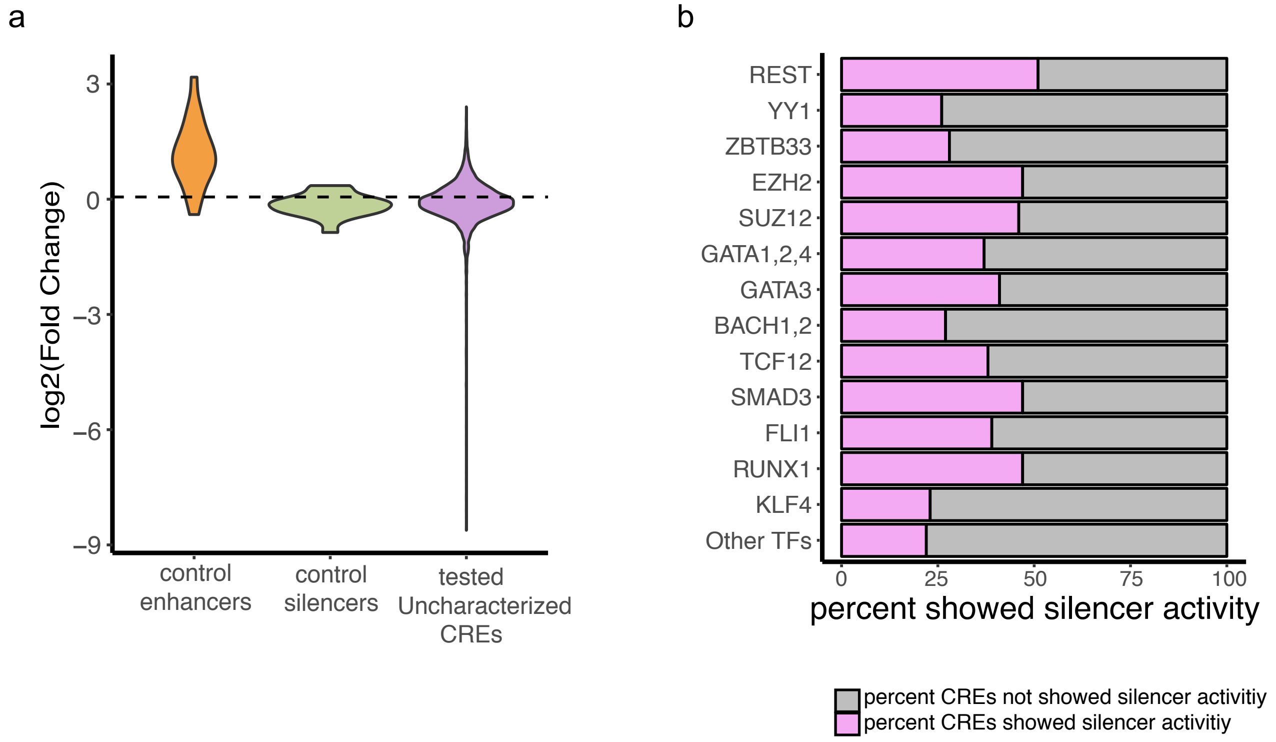

c

de novo motif	known motif	# of silencers	p-val
CTGTCCATGGTG	REST	166	1e-249
AAATGGCT	YY1	34	1e-14
GTTCTCATGAGA	ZBTB33	49	1e-63
ACTGCACGAG	ZBTB3	50	1e-31

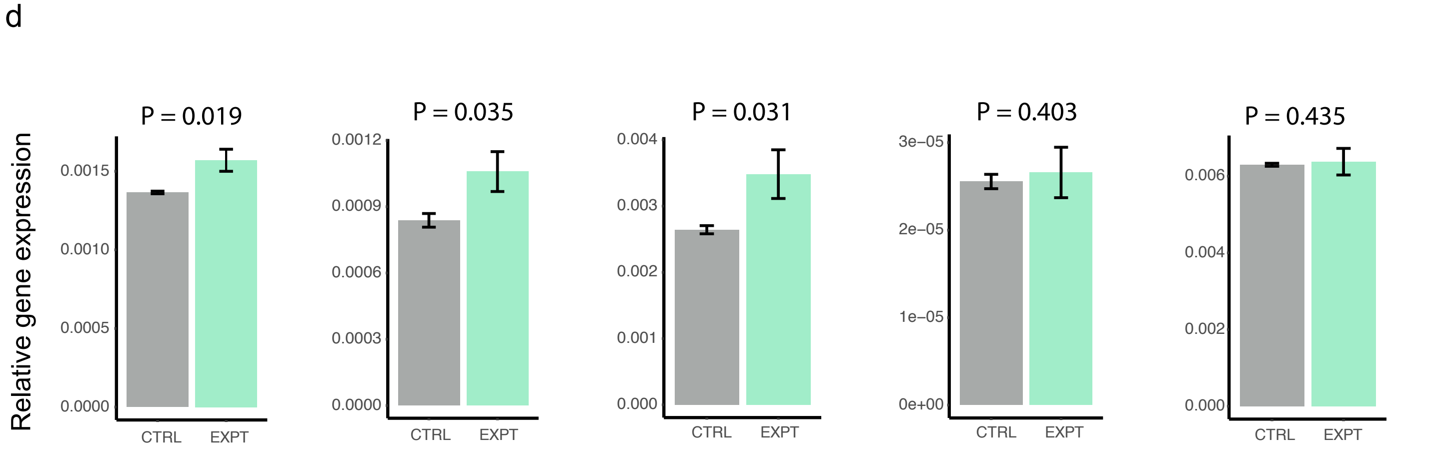

Silencer 7
chr19: 46,442,981 - 46,443,080
Target gene: DMPK

Silencer 8
chr18: 77,352,826 - 77,352,910
Target gene: NFATC1

Silencer 9
chr2: 12,997,131 - 12,997,294
Target gene: TRIB2

Silencer 10
chr15 : 45,213,928 - 45,214,036
Target gene: SHF

Silencer 11
chr12 : 104,556,911 - 104,557,086
Target gene: RP11-650K20.2

Supplementary Figure 2: Functional testing of activity of uncharacterized CREs in K562 cells. (a) Violin plot displaying the activity level distributions of enhancers, control silencers and tested uncharacterized CREs relative to activity of random regions. The dotted horizontal line indicates the mean activity of random regions and acts as a reference. (b) Bar plot showing the fraction of tested uncharacterized CREs categorized by different TFs. (c) Enriched *de novo* motifs related to well-known repressor TFs in validated uncharacterized CREs. (d) Box plots representing relative transcript levels measured by qRT-PCR for candidate target genes for CRISPR edited uncharacterized CREs. Data are represented as 2^(-dCt) relative to internal controls. Error bars represent the mean ± s.d. of biological replicates. It is to be noted that CRISPR experiments are performed in a highly heterogeneous (polyclonal background) population to examine the effects of deletions and used only a single non-targeting control gRNA targeting lambda phage DNA.

Supplementary Figure 3

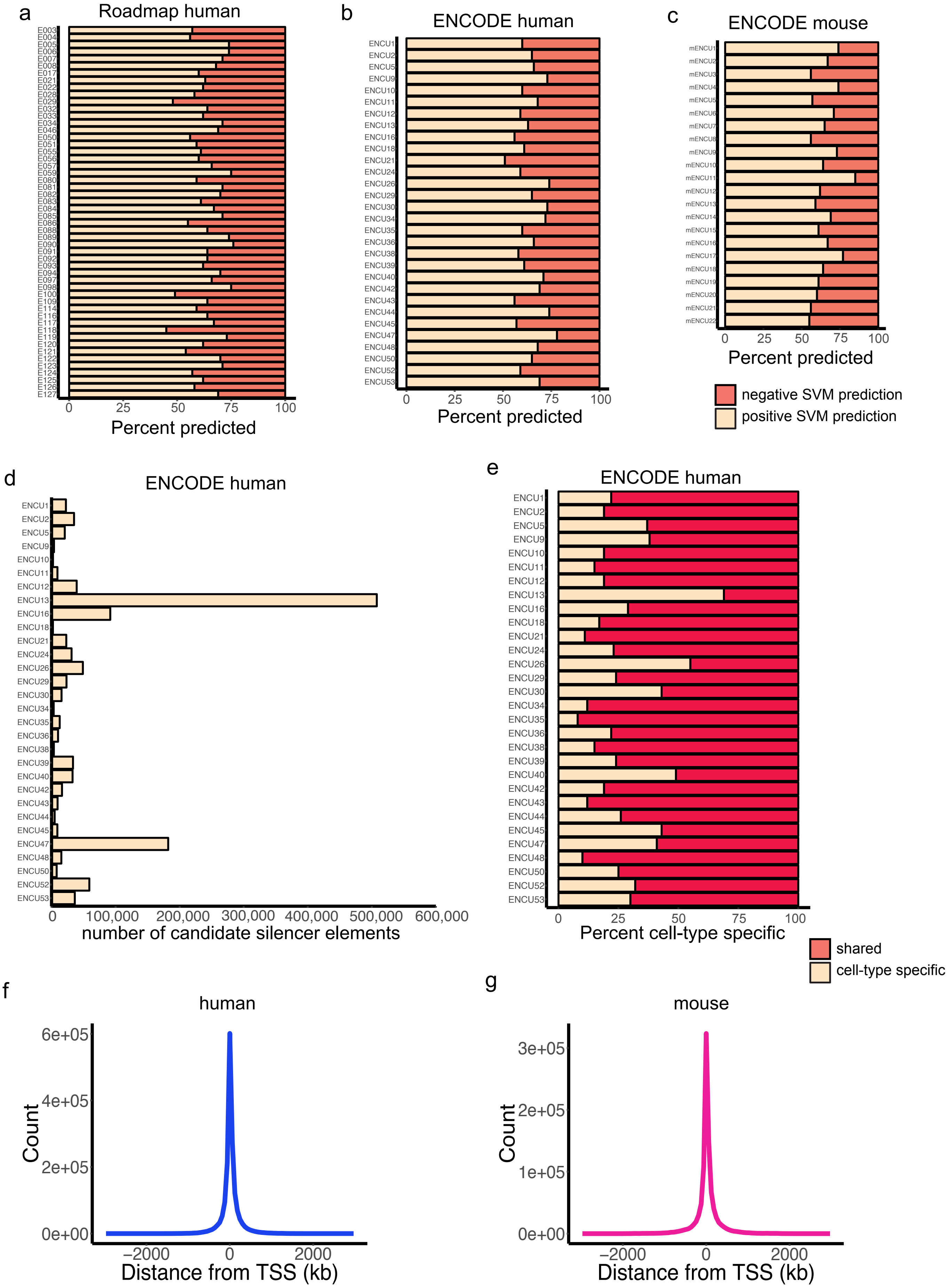

Supplementary Figure 3: SVM model candidate silencer elements predictions. Bar plot showing the fraction of SVM-predicted candidate silencer elements from uncharacterized CREs in 52 human cell types and tissues from Roadmap (a), 30 human cell types and tissues from ENCODE (b) and 22 mouse cell types and tissues from ENCODE (c). (d) Bar plot presenting the count of predicted candidate silencer elements across different cell types and tissues in human from ENCODE. (e) Distribution of cell-type specific candidate silencer elements in human from ENCODE. The location of putative silencers relative to gene TSS in human (f) and mouse (g).

Supplementary Figure 4

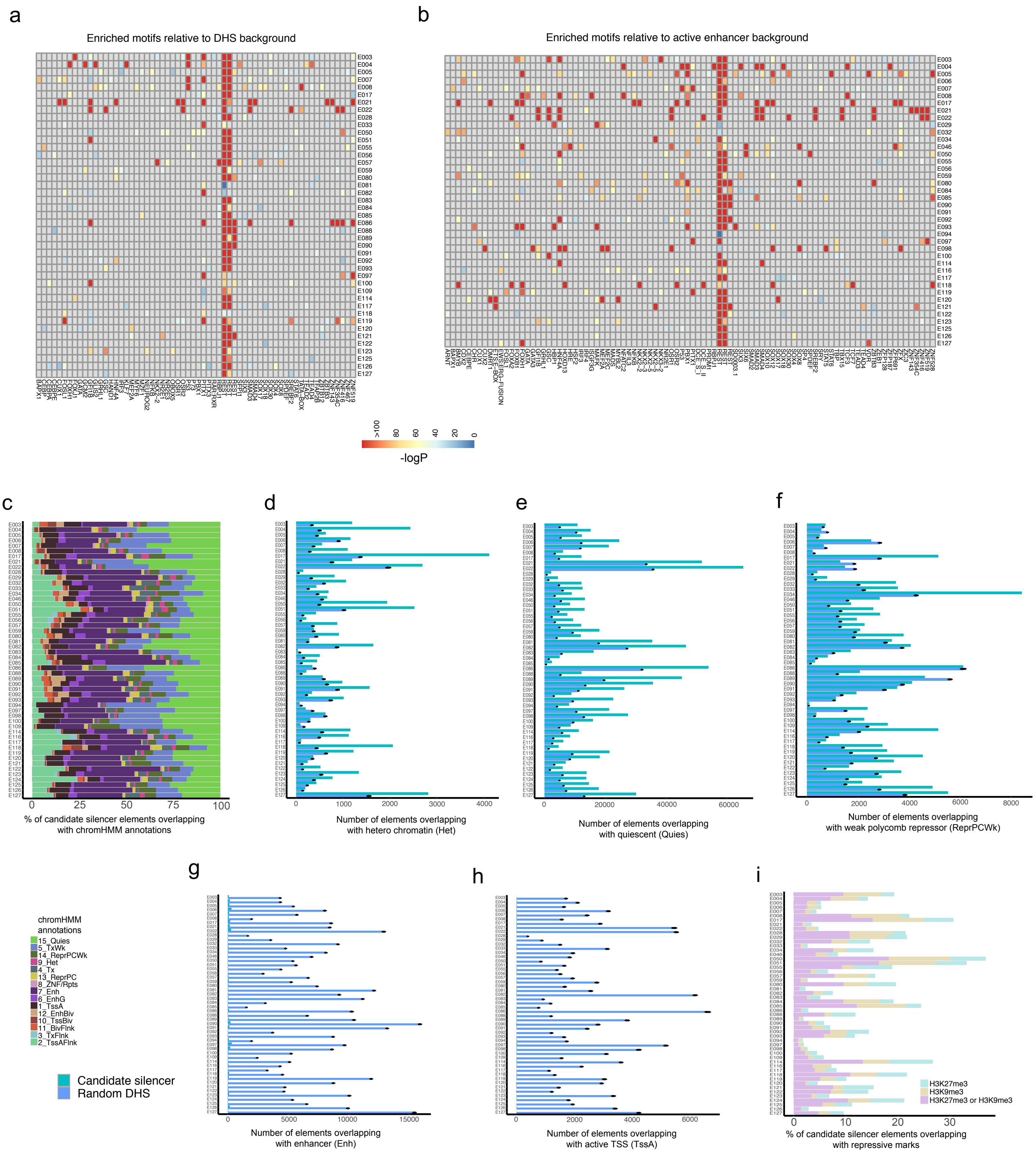

Supplementary Figure 4: Characteristics of candidate silencer elements. Heatmap of enrichment (-log(P-value)) of *de novo* TF motifs and known TF motifs at candidate silencers in comparison to DHS (a) and enhancer backgrounds (b). (c) Bar plot showing the distribution of chromHMM annotations of random DHS elements across 52 cell types in human. The relative number of candidate silencer elements and random DHS at heterochromatin (d), quiescent (e), weak polycomb repressor (f), enhancer (g) and active TSS (h). (i) The percent of candidate silencer elements overlapping with H3K27me3 and/or H3K9me3. Error bars represent the mean ± s.d. of 10000 permuted simulation data.

Supplementary Figure 5

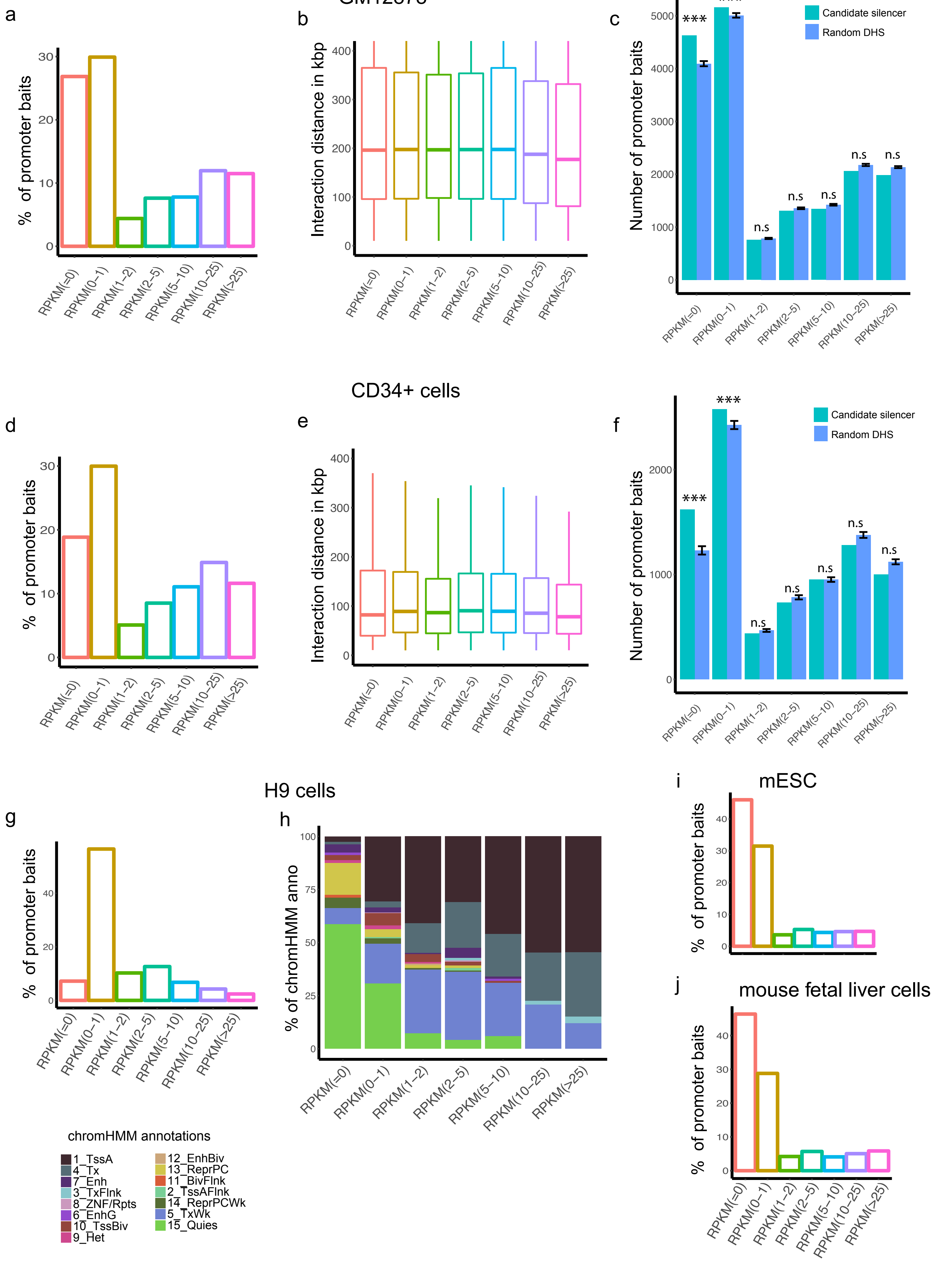

Supplementary Figure 5: 3D Genome interactions of candidate silencer elements. Bar plot presenting the percent of total genes expressed at different expression levels (RPKM) interacting with candidate silencer elements in GM12878 cells (a), CD34+ cells (d), H9 cells (g), mESCs (i), and mouse fetal liver cells (j). Box plot showing the distribution of interaction distances between gene promoter and silencer elements in GM12878 (b) and CD34+ cells (e). Bar plot presenting the counts of genes expressed at different expression levels (RPKM) interacting with candidate silencer elements and random DHS in GM12878 cells (c) and CD34+ cells (f). *** indicates permutation test P-value < 0.0001 and n.s. denotes not significant. (h) Bar plot showing the distribution of chromHMM annotations for TSS of genes interacting with candidate silencer elements in H9 cells. Error bars represent the mean ± s.d. of 10000 permuted simulation data. In the box plots, bounds of the box spans from 25 to 75% percentile, center line represents median, and whiskers visualize 5 and 95% of the data points.

Supplementary Figure 6

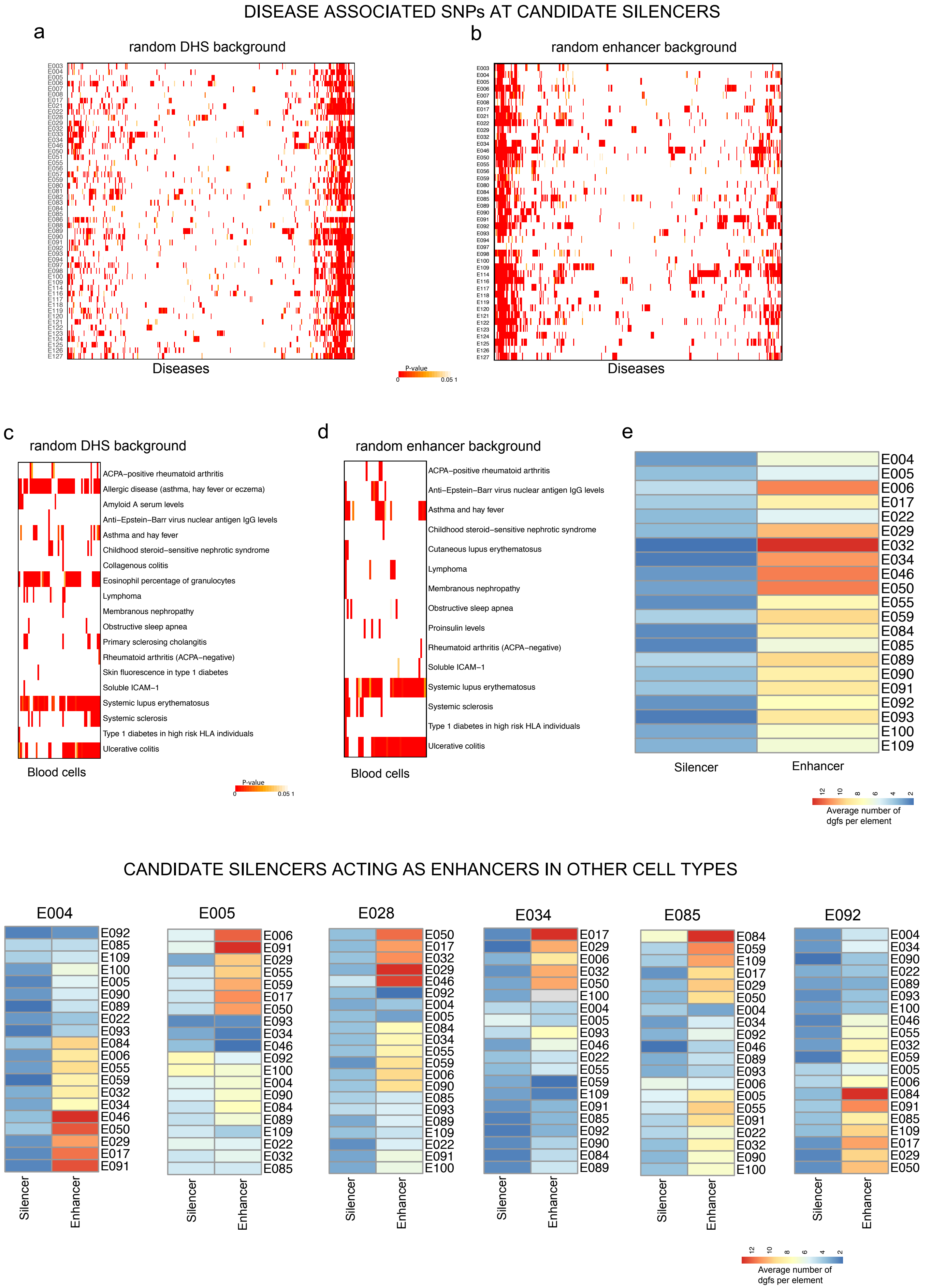

Supplementary Figure 6: Candidate silencer elements are enriched for GWAS SNPs and can act as enhancers in other cell types. Heatmap of enrichment (-log10(P-value)) of 451 disease traits SNPs at candidate silencer elements in comparison to random DHS (a) and random enhancer (b) backgrounds across 52 cell types. Columns represent disease traits and rows are cell types. Heatmap of SNP enrichment (-log10(P-value)) for autoimmune disease traits at candidate silencer elements in comparison to random DHS (c) and random enhancer (d) backgrounds across blood cell types. (e) Heatmap showing the average number of digital genomic footprints (DGF) at candidate silencer elements and enhancers within a noted Roadmap cell type. (f) Heatmaps showing the average number of digital genomic footprints (DGF) at silencer elements acting as silencers in one one cell type (Roadmap number above heatmap) and at silencers acting as enhancers in other cell types (Roadmap number to the right of each row).
