# Supplementary material for: Candidate silencer elements for the human and mouse genomes
Source: Nat Commun. 2020 Feb 26;11:1061. doi: 10.1038/s41467-020-14853-5 (PMC7044160; doi:10.1038/s41467-020-14853-5)
Supplement: Supplementary file 3 — Description of Additional Supplementary Files [file 41467_2020_14853_MOESM3_ESM.pdf]

Description of Additional Supplementary Files

File Name: Supplementary Data 1
Description: Overview of cell types and count of uncharacterized CREs per cell type

File Name: Supplementary Data 2
Description: MPRA tested uncharacterized cis-regulatory elements along with control elements and their activity levels

File Name: Supplementary Data 3
Description: Reporter assays testing silencer elements along with control elements and their activity levels

File Name: Supplementary Data 4
Description: Primer and gblock sequences used in CRISPR-Cas9 experiments

File Name: Supplementary Data 5
Description: SVM model identified candidate silencer elements
